# Fatal H5N6 Avian Influenza Virus Infection in a Domestic Cat and Wild Birds in China

**DOI:** 10.1038/srep10704

**Published:** 2015-06-02

**Authors:** Zhijun Yu, Xiaolong Gao, Tiecheng Wang, Yanbing Li, Yongcheng Li, Yu Xu, Dong Chu, Heting Sun, Changjiang Wu, Shengnan Li, Haijun Wang, Yuanguo Li, Zhiping Xia, Weishi Lin, Jun Qian, Hualan Chen, Xianzhu Xia, Yuwei Gao

**Affiliations:** 1 Research Center of Wildlife Disease, Key Laboratory of Jilin Province for Zoonosis Prevention and Control, Military Veterinary Research Institute of Academy of Military Medical Sciences, Changchun, Jilin Province 130122, People’s Republic of China; 2 Institute of Laboratory Animal Sciences, Chinese Academy of Medical Sciences & Peking Union Medical College, Beijing 100021, People’s Republic of China; 3 State Key Laboratory of Veterinary Biotechnology, Harbin Veterinary Research Institute, Chinese Academy of Agricultural Sciences, Harbin 150001, People’s Republic of China; 4 General Station for Surveillance of Wildlife Diseases & Wildlife Borne Disease, State Forestry Administration, Shenyang, 110034, People’s Republic of China; 5 College of Animal Science and Veterinary Medicine, Jilin Agricultural University, Changchun, 130118, People’s Republic of China; 6 College of Veterinary Medicine, Gansu Agricultural University, Lanzhou, 730070, People’s Republic of China; 7 Changchun Veterinary Research Institute, Chinese Academy of Agricultural Sciences, Changchun 130122, People’s Republic of China; 8 Jiangsu Co-innovation Center for Prevention and Control of Important Animal Infectious Diseases and Zoonoses, Yangzhou, 225009, People’s Republic of China

## Abstract

H5N6 avian influenza viruses (AIVs) may pose a potential human risk as suggested by the first documented naturally-acquired human H5N6 virus infection in 2014. Here, we report the first cases of fatal H5N6 avian influenza virus (AIV) infection in a domestic cat and wild birds. These cases followed human H5N6 infections in China and preceded an H5N6 outbreak in chickens. The extensive migration routes of wild birds may contribute to the geographic spread of H5N6 AIVs and pose a risk to humans and susceptible domesticated animals, and the H5N6 AIVs may spread from southern China to northern China by wild birds. Additional surveillance is required to better understand the threat of zoonotic transmission of AIVs.

On May 7, 2014, China formally confirmed the first human infection with an H5N6 avian influenza virus (AIV)^1^,^2^. The patient, a 49-year-old male in Sichuan province, was hospitalized with severe pneumonia and died. Notably, the identification of the first human case of H5N6 virus infection coincided with reports of human infections with H7N9 and H10N8 AIVs in China^3^,^4^,^5^. H7N9 and H10N8 AIVs have also been shown to infect some animals, including feral dogs^6^ and tree sparrows^7^, suggesting that mammals and wild birds may play a role in the ongoing circulation of novel AIVs that sporadically infect humans in China. Here, we report the first cases of fatal H5N6 AIV infection in a domestic cat and wild birds.

## Results

### Phylogenetic Analysis

**Figure 1:**
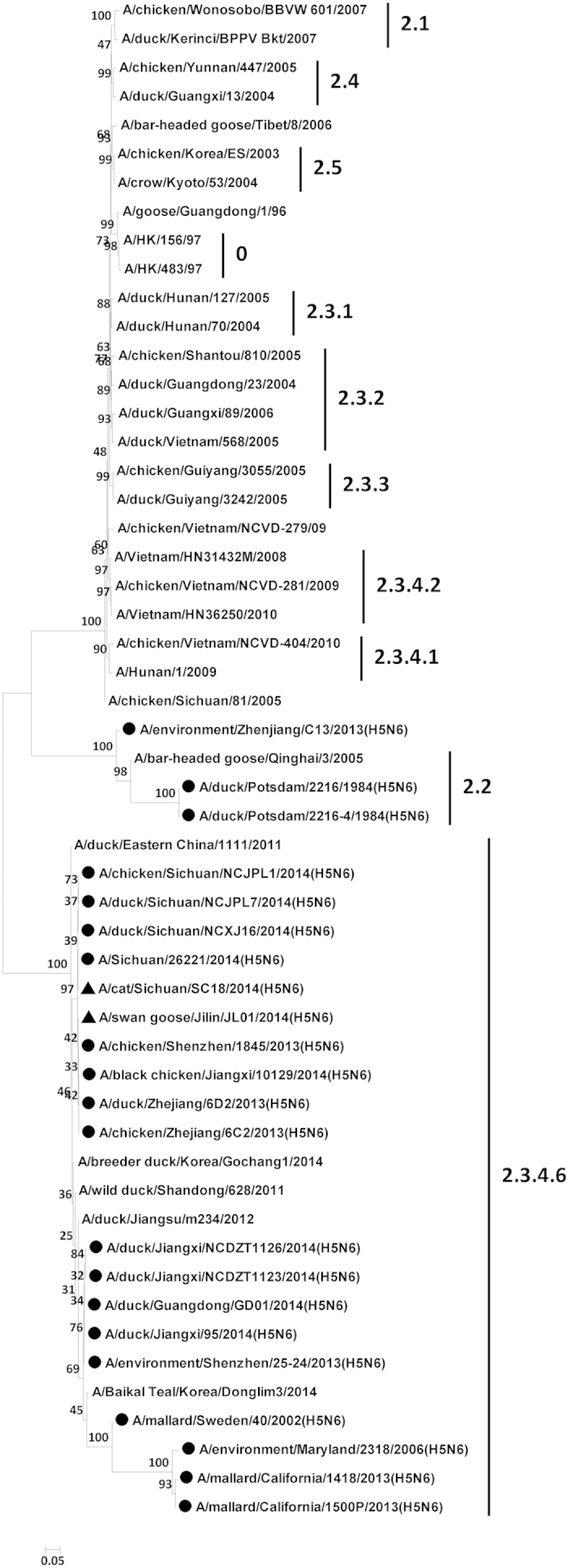
Phylogenetic tree of the hemagglutinin (HA) genes of influenza A (H5N6) viruses, China, 2014. Triangles indicate viruses characterized in this study. Other H5N6 viruses are indicated in circles. Horizontal branch lengths are proportional to genetic distances. Scale bar indicates nucleotide substitutions per site.

The lung specimen from the deceased cat and all lung specimens from three deceased swan geese were positive for H5N6 AIV. No other influenza virus subtypes were detected. The viral genomes of each isolate were sequenced to assess the molecular and epidemiological characteristics of these H5N6 strains. The PB2, PB1, PA, HA, NP, NA, M, and NS segments of A/cat/Sichuan/SC18/2014 (H5N6) show 99.5%, 99.5%, 99.3%, 99.4%, 97.3%, 98.9%, 99.9%, and 99.6% nucleotide sequence identity to the corresponding segments of A/swan goose/Jilin/JL01/2014 (H5N6). Phylogenetic analysis of the HA gene sequences indicated that the HA segments of each isolate were derived from the Asian H5N1 clade 2.3.4.6 HA lineage (Fig. 1). Collectively, the gene segments of A/cat/Sichuan/SC18/2014 and A/swan goose/Jilin/JL01/2014 were most closely related to a human H5N6 influenza isolate named A/Sichuan/26221/2014 (Table 1). Phylogenetic analyses suggest that the newly isolated H5N6 viruses possess high nucleotide sequence identity to H5N6 AIV subtypes previously isolated in southern China (Fig. 1 and Figure S1-S7). We speculate that the H5N6 virus may have emerged in Sichuan province in early 2014, and subsequently spread to Jilin provinces through the migration of wild birds.

### Analysis of molecular features associated with AIV virulence, transmissibility, and antiviral resistance

We next analyzed whether these H5N6 strains possessed key molecular features associated with increased virulence in mammals, mammalian transmissibility, and antiviral resistance (Table 2). The H5N6 viruses displayed some molecular markers associated with increased virulence and transmission in mammals. A/cat/Sichuan/SC18/2014 (H5N6) and A/swan goose/Jilin/JL01/2014 (H5N6) possessed an alanine at position 160 which is associated with H5 transmissibility in ferrets, and an aspartic acid residue at M1 position 30, an alanine residue at M1 position 215, and a serine residue at NS1 position 42 which are associated with increased virulence in mice (Table 2). Several studies have suggested that NA stalk truncation enhances H5N1 virulence in mice and increases replication and virulence of AIVs in chicken eggs and poultry^8^,^9^,^10^. A deletion of amino acids 59-69 of the NA stalk has been reported in some H5N6 viruses, although no such stalk deletion was found in A/cat/Sichuan/SC18/2014 (H5N6) and A/swan goose/Jilin/JL01/2014 (H5N6). Molecular markers of oseltamivir- and amandatide-resistance were also not present in the NA and M1 protein sequences of the H5N6 viruses, indicating that they should be still sensitive to influenza antiviral drugs.

## Discussion

In this study, we provide the first evidence of fatal H5N6 AIV infection in domestic cats and wild birds. The extensive migration routes of wild birds may contribute to the geographic spread of H5N6 AIVs and pose a risk to humans and susceptible domesticated animals. Based on our phylogenetic analysis, we speculated that the H5N6 AIVs may spread from southern China to northern China by wild birds. This hypothesis is supported by the recent outbreak of H5N6 AIV among chickens in Harbin city, Heilongjiang province, China, which affected 20,550 chickens of which 17,790 died^11^. The H5N6-infected swan geese were identified in Baicheng, China which is adjacent to inner Mongolia. The middle migratory flyway of China includes inner Mongolia to the north and Sichuan province to the south^12^. Wild birds in or near Baicheng could migrate to Sichuan province via this path. Additionally, documentation of H5N6 infection of domestic cats raises the possibility that cats could play a role in the mammalian adaptation or perhaps transmission of novel avian influenza viruses. Therefore, continuous epidemiological monitoring of different animal species such as cats, dogs, and wild birds should be performed to effectively reduce the threat of zoonotic transmission of influenza strains like the newly emergent H5N6 avian influenza virus.

## Methods

### Ethics statement

The protocol of the study was conducted in accordance with guidelines of animal welfare of World Organization for Animal Health. All experimental protocol were approved by the Review Board Military Veterinary Research Institute of the Academy of Military Medical Sciences.

### Facilities

Studies with H5N6 AIVs were conducted in a biosecurity level 3 laboratory approved by the Military Veterinary Research Institute of the Academy of Military Medical Sciences.

### Sample Collection

During May–June 2014, seventy feces specimens from wild birds and one lung specimen from a dead domestic cat were collected in areas with close-proximity to the residence of the patient infected with H5N6 AIV in Nanchong city, Sichuan province, southwest China. The domestic cat is suspected of having succumbed to AIV infection based on flu-like clinical signs and necropsy findings. Additionally, lung specimens were collected following the sudden and unexplained deaths of three swan geese in Baicheng city, Jilin province, northeast China. No additional information was available. We screened all specimens by RT-PCR for evidence of influenza virus infection.

### Virus Isolation

We inoculated the allantoic cavities of 10-day-old specific pathogen-free embryonated chicken eggs with material from the H5N6 AIV-positive lung specimen from the domestic cat and one swan goose. After 60 hours incubation at 37 °C, we recovered two virus isolates, named A/cat/Sichuan/SC18/2014 (H5N6) and A/swan goose/Jilin/JL01/2014 (H5N6).

### RNA isolation, PCR amplification, and Sequencing

Viral RNA was isolated from the allantoic fluid of inoculated eggs using the RNeasy Mini kit (QIAGEN, Germantown, MD) according to the manufacturer’s protocol. Reverse transcription of viral RNA and subsequent PCR were performed using primers specific for each gene segment (sequences available upon request). PCR products were purified using the QIAquick PCR purification kit (QIAGEN, Germantown, MD) according to the manufacturer’s protocol. Viral gene segments were sequenced by the Beijing Genomics Institute (Beijing, China). The GenBank accession numbers of A/cat/Sichuan/SC18/2014 (H5N6) and A/swan goose/Jilin/JL01/2014 (H5N6) are KM873635 to KM873650, respectively.

### Phylogenetic Analysis

To investigate the molecular and epidemiological characteristics and to determine the profile of genetic diversity, phylogenetic trees were constructed using molecular evolutionary genetics analysis MEGA 6 (http://www.megasoftware.net/mega.php) with the neighbor-joining (NJ) method to calculate distance. Bootstrap values were estimated for 1,000 replicates.

## Additional Information

**How to cite this article**: Yu, Z. *et al.* Fatal H5N6 Avian Influenza Virus Infection in a Domestic Cat and Wild Birds in China. *Sci. Rep.* **5**, 10704; doi: 10.1038/srep10704 (2015).

## Supplementary Material

Supplementary Information

## Figures and Tables

**Table 1:** Levels of nucleotide sequence identity between A/cat/Sichuan/SC18/2014 (H5N6) or A/swan goose/Jilin/JL01/2014 (H5N6) and A/Sichuan/26221/2014 (H5N6).

Virus	Gene*	Virus with the highest percentage of nucleotide identity	GISAID accession no.	Identity, %
A/cat/Sichuan/SC18/2014 (H5N6)	PB2	A/Sichuan/26221/2014(H5N6)	EPI533585	99.9
	PB1	A/Sichuan/26221/2014(H5N6)	EPI533586	100.0
	PA	A/Sichuan/26221/2014(H5N6)	EPI533587	99.9
	HA	A/Sichuan/26221/2014(H5N6)	EPI533583	100.0
	NP	A/Sichuan/26221/2014(H5N6)	EPI533588	99.9
	NA	A/Sichuan/26221/2014(H5N6)	EPI533584	99.8
	M	A/Sichuan/26221/2014(H5N6)	EPI533589	99.9
	NS	A/Sichuan/26221/2014(H5N6)	EPI533590	100.0
A/swan goose/Jilin/JL01/2014 (H5N6)	PB2	A/Sichuan/26221/2014(H5N6)	EPI533585	99.6
	PB1	A/Sichuan/26221/2014(H5N6)	EPI533586	99.5
	PA	A/Sichuan/26221/2014(H5N6)	EPI533587	99.4
	HA	A/Sichuan/26221/2014(H5N6)	EPI533583	99.4
	NP	A/Sichuan/26221/2014(H5N6)	EPI533588	97.4
	NA	A/Sichuan/26221/2014(H5N6)	EPI533584	99.1
	M	A/Sichuan/26221/2014(H5N6)	EPI533589	99.8
	NS	A/Sichuan/26221/2014(H5N6)	EPI533590	99.6

^*^PB2, basic polymerase 2; PB1, basic polymerase 1; PA, acidic polymerase; HA, hemagglutinin; NP, nucleoprotein; NA, neuraminidase; M, matrix; NS, nonstructural protein.

**Table 2:**
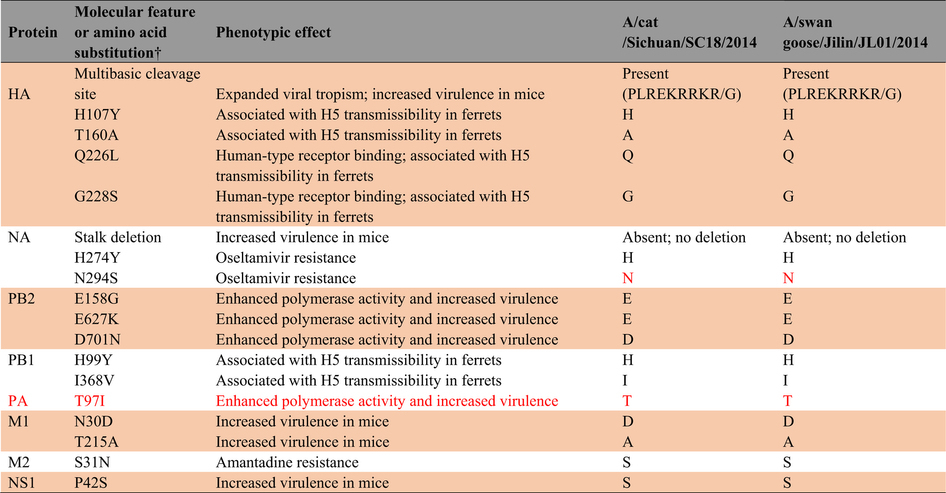
Analysis of molecular features associated with AIV virulence, transmissibility, and antiviral resistance in H5N6 AIV isolates from a domestic cat and swan goose*
