# Supplementary material for: Fatal H5N6 Avian Influenza Virus Infection in a Domestic Cat and Wild Birds in China
Source: Sci Rep. 2015 Jun 2;5:10704. doi: 10.1038/srep10704 (PMC4603707; doi:10.1038/srep10704)
Supplement: Supplementary Information [file srep10704-s1.pdf]

Supplementary Information

Fatal H5N6 Avian Influenza Virus Infection in a Domestic Cat and Wild Birds in China

Zhijun Yu, Xiaolong Gao, Tiecheng Wang, Yanbing Li, Yongcheng Li, Yu Xu, Dong Chu, Heting Sun, Changjiang Wu, Shengnan Li, Haijun Wang, Yuanguo Li, Zhiping Xia, Weishi Lin, Jun Qian, Hualan Chen, Xianzhu Xia & Yuwei Gao

Supplementary Figure S1

Supplementary Figure S2

Supplementary Figure S3

Supplementary Figure S4

Supplementary Figure S5

Supplementary Figure S6

Supplementary Figure S7

Figure S1

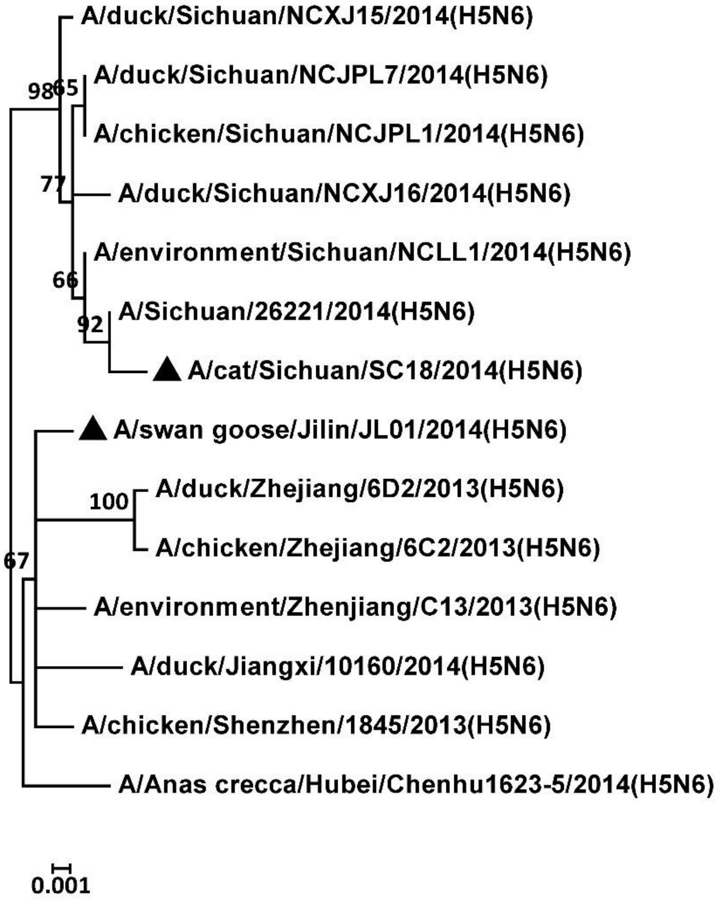

Figure S1. Phylogenetic tree of the hemagglutinin (NA) genes of influenza A (H5N6) viruses, China, 2014. Triangles indicate viruses characterized in this study. Horizontal branch lengths are proportional to genetic distances. Scale bar indicates nucleotide substitutions per site.

Figure S2

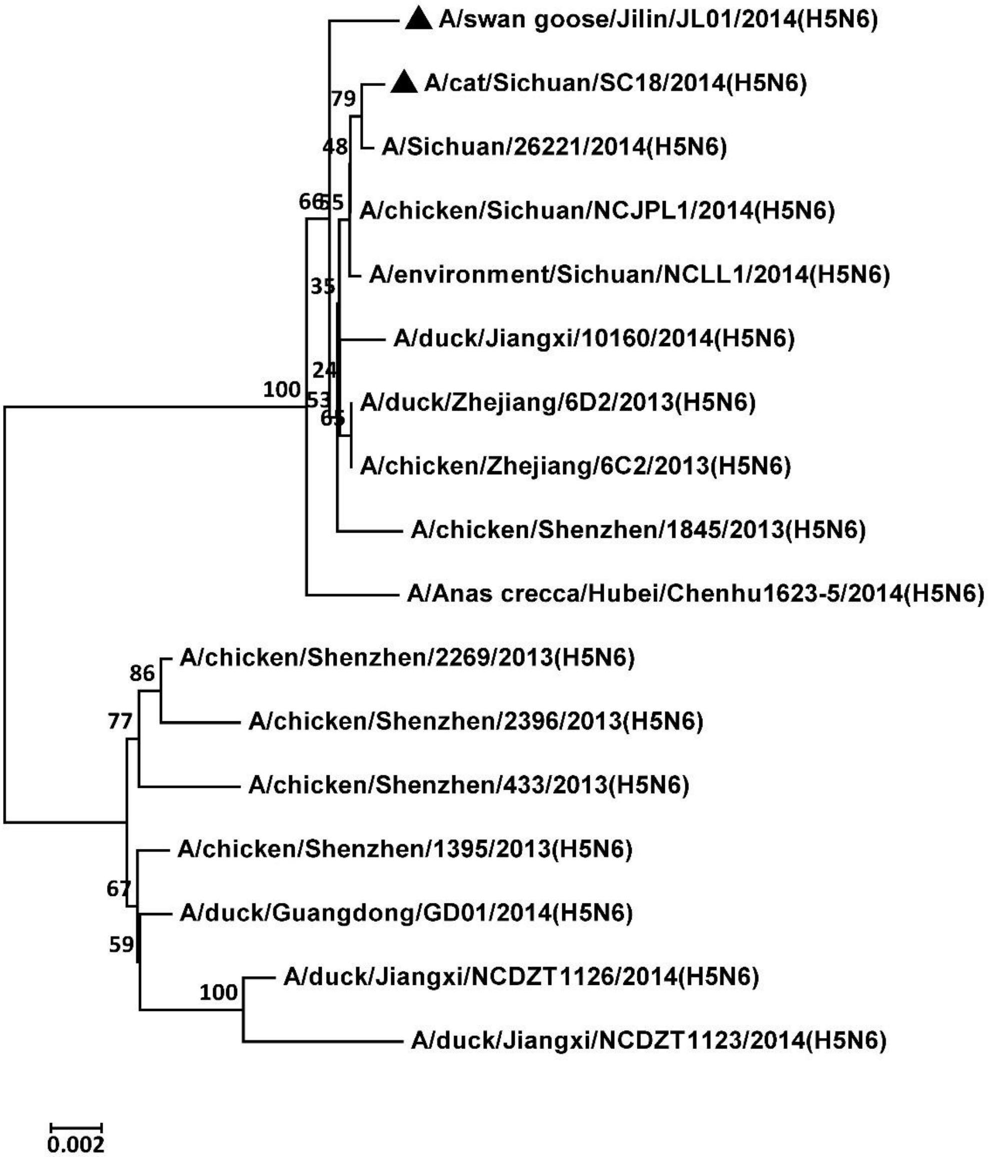

Figure S2. Phylogenetic tree of the hemagglutinin (PB2) genes of influenza A (H5N6) viruses, China, 2014. Triangles indicate viruses characterized in this study. Horizontal branch lengths are proportional to genetic distances. Scale bar indicates nucleotide substitutions per site.

Figure S3

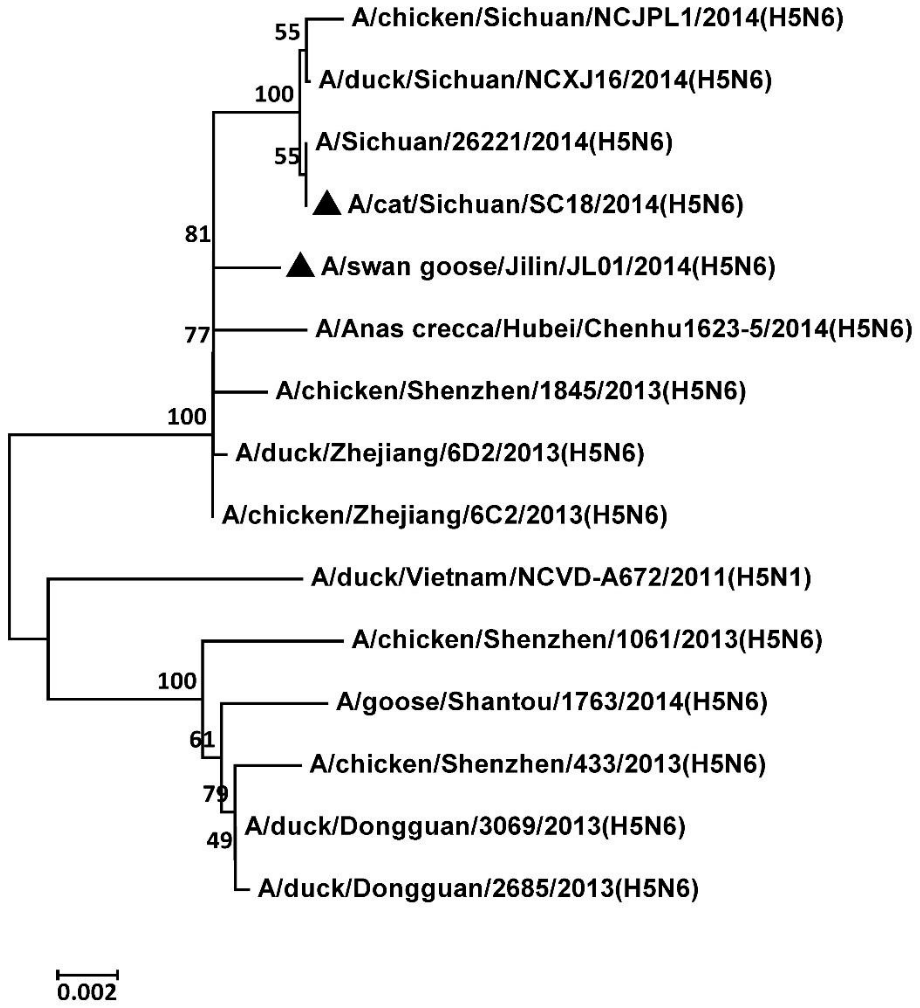

Figure S3. Phylogenetic tree of the hemagglutinin (PB1) genes of influenza A (H5N6) viruses, China, 2014. Triangles indicate viruses characterized in this study. Horizontal branch lengths are proportional to genetic distances. Scale bar indicates nucleotide substitutions per site.

Figure S4

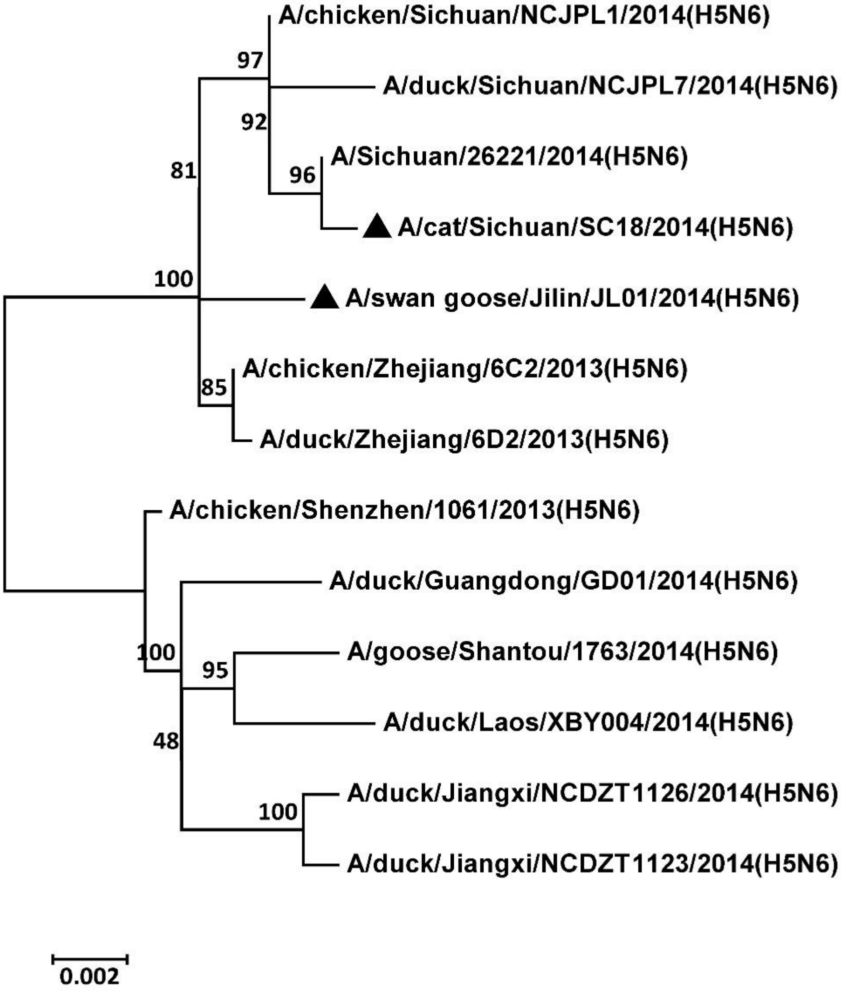

Figure S4. Phylogenetic tree of the hemagglutinin (PA) genes of influenza A (H5N6) viruses, China, 2014. Triangles indicate viruses characterized in this study. Horizontal branch lengths are proportional to genetic distances. Scale bar indicates nucleotide substitutions per site.

Figure S5

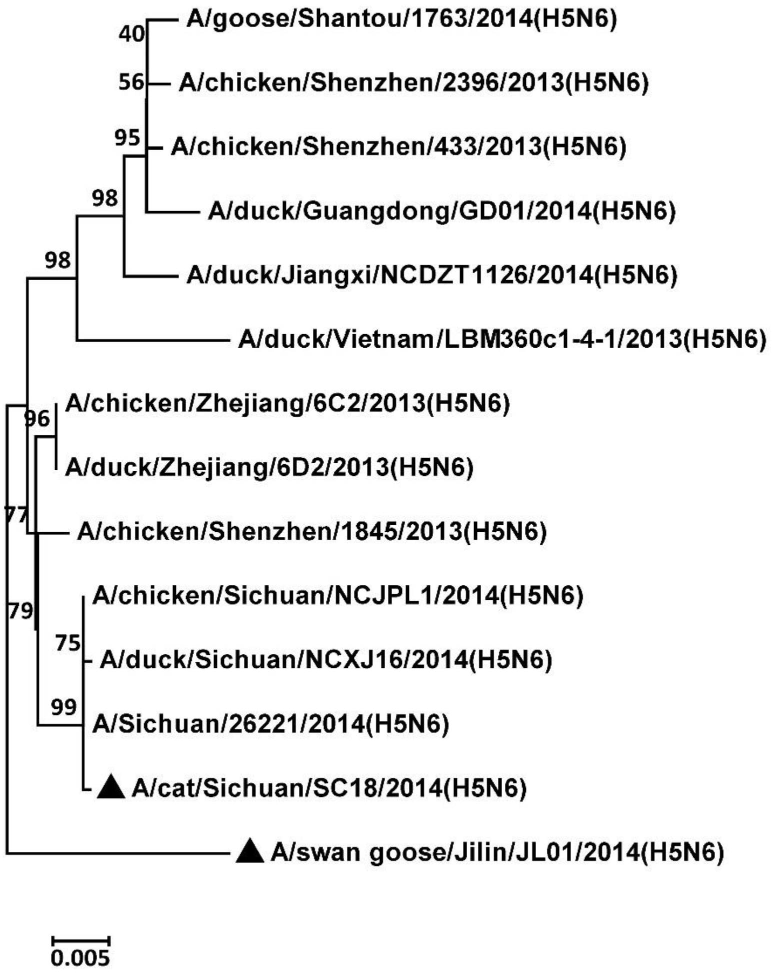

Figure S5. Phylogenetic tree of the hemagglutinin (NP) genes of influenza A (H5N6) viruses, China, 2014. Triangles indicate viruses characterized in this study. Horizontal branch lengths are proportional to genetic distances. Scale bar indicates nucleotide substitutions per site.

Figure S6

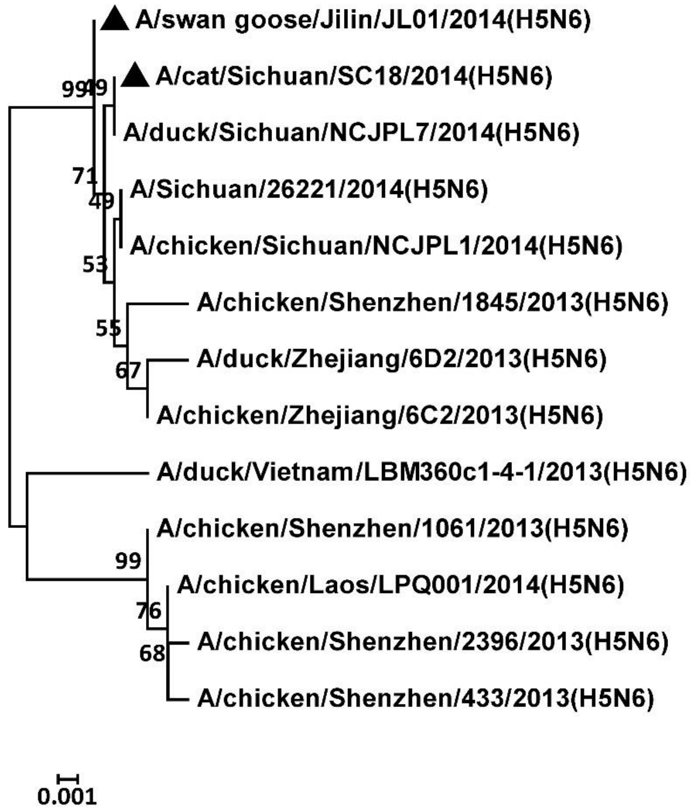

Figure S6. Phylogenetic tree of the hemagglutinin (M) genes of influenza A (H5N6) viruses, China, 2014. Triangles indicate viruses characterized in this study. Horizontal branch lengths are proportional to genetic distances. Scale bar indicates nucleotide substitutions per site.

Figure S7

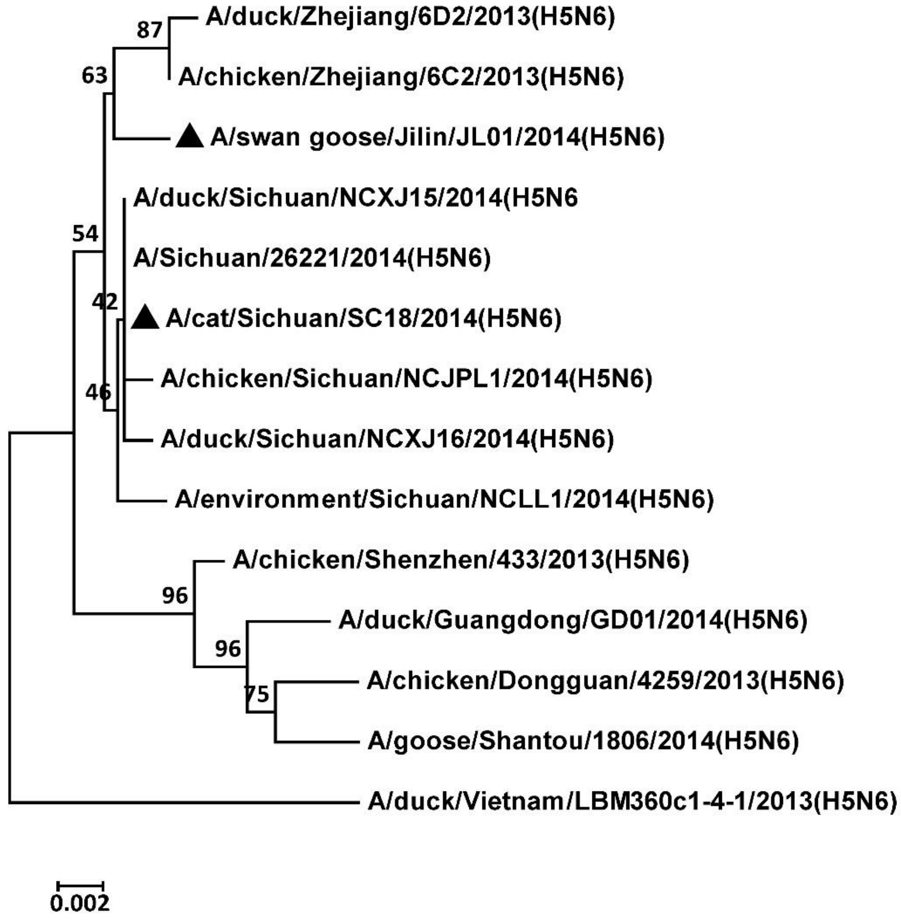

Figure S7. Phylogenetic tree of the hemagglutinin (NS) genes of influenza A (H5N6) viruses, China, 2014. Triangles indicate viruses characterized in this study. Horizontal branch lengths are proportional to genetic distances. Scale bar indicates nucleotide substitutions per site.
